# Surveillance for Yellow Fever Virus in Non-Human Primates in Southern Brazil, 2001–2011: A Tool for Prioritizing Human Populations for Vaccination

**DOI:** 10.1371/journal.pntd.0002741

**Published:** 2014-03-13

**Authors:** Marco A. B. Almeida, Jader da C. Cardoso, Edmilson dos Santos, Daltro F. da Fonseca, Laura L. Cruz, Fernando J. C. Faraco, Marilina A. Bercini, Kátia C. Vettorello, Mariana A. Porto, Renate Mohrdieck, Tani M. S. Ranieri, Maria T. Schermann, Alethéa F. Sperb, Francisco Z. Paz, Zenaida M. A. Nunes, Alessandro P. M. Romano, Zouraide G. Costa, Silvana L. Gomes, Brendan Flannery

**Affiliations:** 1 Division of Environmental Health Surveillance, Health Surveillance Coordination, Rio Grande do Sul State Health Department, Porto Alegre, Rio Grande do Sul, Brazil; 2 La Salle University, Canoas, Rio Grande do Sul, Brazil; 3 Division of Epidemiologic Surveillance, Health Surveillance Coordination, Rio Grande do Sul State Health Department, Porto Alegre, Rio Grande do Sul, Brazil; 4 Health Surveillance Coordination, Rio Grande do Sul State Health Department, Porto Alegre, Rio Grande do Sul, Brazil; 5 Central State Public Health Laboratory, Rio Grande do Sul State Health Department, Porto Alegre, Rio Grande do Sul, Brazil; 6 Secretariat for Health Surveillance, Brazilian Ministry of Health, Brasília, Brazil; 7 Pan American Health Organization, Brasília, Brazil; 8 Global Immunization Division, Center for Global Health, U.S. Centers for Disease Control and Prevention, Atlanta, Georgia, United States of America; Duke-NUS, Singapore

## Abstract

In Brazil, epizootics among New World monkey species may indicate circulation of yellow fever (YF) virus and provide early warning of risk to humans. Between 1999 and 2001, the southern Brazilian state of Rio Grande do Sul initiated surveillance for epizootics of YF in non-human primates to inform vaccination of human populations. Following a YF outbreak, we analyzed epizootic surveillance data and assessed YF vaccine coverage, timeliness of implementation of vaccination in unvaccinated human populations. From October 2008 through June 2009, circulation of YF virus was confirmed in 67 municipalities in Rio Grande do Sul State; vaccination was recommended in 23 (34%) prior to the outbreak and in 16 (24%) within two weeks of first epizootic report. In 28 (42%) municipalities, vaccination began more than two weeks after first epizootic report. Eleven (52%) of 21 laboratory-confirmed human YF cases occurred in two municipalities with delayed vaccination. By 2010, municipalities with confirmed YF epizootics reported higher vaccine coverage than other municipalities that began vaccination. In unvaccinated human populations timely response to epizootic events is critical to prevent human yellow fever cases.

## Author Summary

Yellow fever (YF) is a viral hemorrhagic disease that affects humans as well as several species of non-human primates, especially New World monkeys found in South America. Yellow fever virus (YFV) is maintained in a natural cycle involving tree-hole breeding mosquitoes and non-human primates hosts. Because YF is often fatal in susceptible New World monkey populations, sudden die-offs of New World monkeys or epizootics can signal YFV circulation in an environment where humans may be exposed. Surveillance for such events is an important tool to prevent human disease. The state of Rio Grande do Sul in southern Brazil was one of the first states to conduct passive and active surveillance for yellow fever virus circulation in non-human primates. During a rapidly-spreading YF outbreak in 2008–2009, surveillance for epizootics involving non-human primates informed vaccination of human populations in areas previously free of YF. In this study, we analyzed surveillance data to evaluate vaccine coverage, timeliness of notification, investigation and confirmation of epizootic events in relation to occurrence of human cases of YF. Epizootic surveillance is useful for directing the efforts of vaccine distribution. Prevention of YF in susceptible human populations requires immediate risk communication and implementation of vaccine recommendations after the first reports of epizootic activity when YF is suspected.

## Introduction

Yellow fever (YF) is a disease caused by yellow fever virus (YFV), a member of the *Flaviviridae* family. The disease is considered endemic in parts of Africa and South America. Due to its varied clinical presentation and limited surveillance, yellow fever is under-reported: annually, approximately 5,000 cases are reported to WHO (World Health Organization) from Africa and 300 cases from South America but true incidence is believed to be 10–50 fold higher [1]. The natural transmission cycle of YF involves tree-hole breeding mosquitoes and a wide array of monkeys, apes and marmosets [1]. While primate species in Africa rarely develop fatal disease following YFV infection [2], several species of New World monkeys in the Americas are susceptible to severe, fatal YF disease. In the Americas, deaths of susceptible non-human primates are sentinel events that may indicate presence of YFV in a specific geographic location or environment and surveillance for such events is an important tool to prevent human disease [3]. The goal of surveillance for deaths among non-human primates is to provide an early warning of risk of YFV transmission to humans, for rapid implementation of vaccination and prevention strategies. However, there have been few reports on how epizootic surveillance has been used to inform YF vaccination in human populations.

In Brazil, YF caused major urban epidemics and was considered a public health scourge in Brazilian cities until successful vector control and human vaccination strategies eliminated urban transmission in 1942 [4]. However, the existence of a sylvatic cycle of YFV transmission involving non-human primates was described in the 1930s [5]. Since that time, sporadic human cases and outbreaks have resulted from human activity in endemic areas and expansion of viral transmission to previously unaffected regions. The coastal area of Brazil is generally considered free of YF, including states on the northern, southeastern and southern coasts [2]. Mainly in rural areas, the sylvatic cycle has been maintained by transmission among non-human primates and mosquito vectors. Some New World monkeys, *e.g.* howler monkeys (genus *Alouatta*) are extremely susceptible to YFV [6] and develop fatal disease, similar to humans [1]. There are records of epizootics characterized by substantial mortality in howler monkeys populations due to this disease [7]–[13].

Surveillance for YF detection in non-human primates was initiated in the state of Rio Grande do Sul, Brazil in 1999, initially as a passive strategy. Active surveillance began in 2001 following detection of YFV in the northwestern part of the state (bordering Argentina) [11], [14], [15] and vaccination of residents in surrounding municipalities. Between December, 2002 and September, 2008, no evidence was found of YFV circulation or of immunity to YFV in blood specimens from more than 200 captured howler monkeys of *Alouatta* species or evidence of YFV in more than 2,000 mosquitoes (including *Haemagogus leucocelaenus*) [16]. Seven years after active surveillance began, a rapidly-spreading YF outbreak in the state between October, 2008 and June, 2009 [7], [17]. The coincident YF epizootic in non-human primates was the largest ever documented, with more than two-thirds of specimens from non-human primates testing positive for YFV infection [7], [17].

The YF outbreak provided the opportunity to evaluate surveillance for epizootic events involving deaths of non-human primates. We assessed timeliness of notification of non-human primate deaths in relation to the occurrence of confirmed human cases, vaccine coverage and timing of YF vaccination in municipalities in the state of Rio Grande do Sul during the YF outbreak.

## Methods

### Ethics statement

This study involved analysis of routinely collected surveillance data and did not require ethical review according to the Brazilian National Committee for Ethics in Research.

Personally identifiable information was available only to surveillance officers and was not used in this study.

### Setting

**Figure 1:**
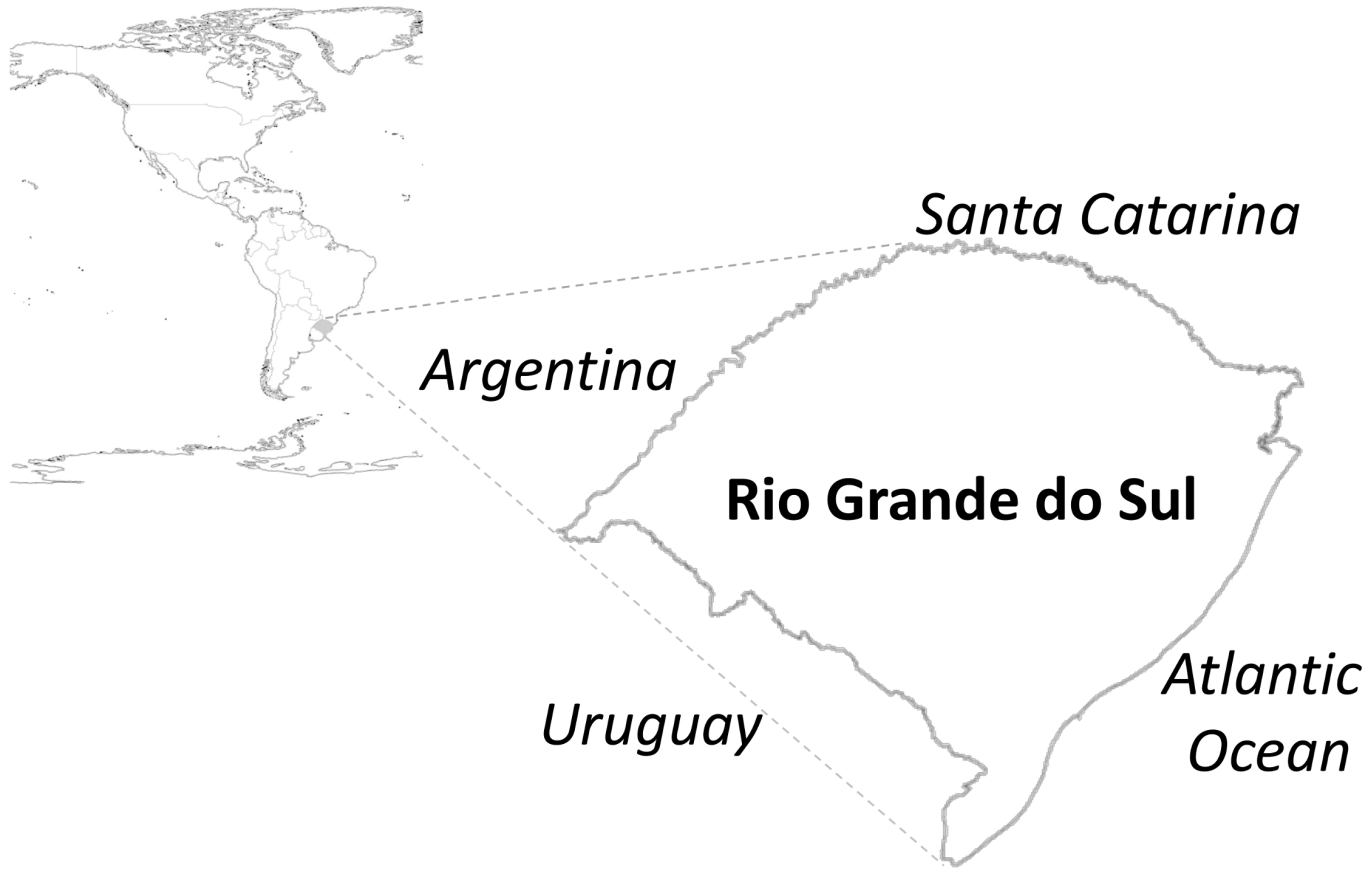
Map showing the state of Rio Grande do Sul, Brazil. The Brazilian state of Santa Catarina lies to the north, Uruguay to the south, Argentina to the west and the Atlantic Ocean to the east.

The state of Rio Grande do Sul is the southernmost state in Brazil, bordering Uruguay to the south and Argentina to the west (Figure 1). It occupies an area of 268,782 km^2^ and has 10,695,532 inhabitants [18]. The state is divided in 496 municipalities, and the capital is Porto Alegre (1,409,939 inhabitants).

### Surveillance for YF in non-human primates

An epizootic is defined as a disease that appears as new cases in a given animal population over a short period of time in a defined geographical area. Yellow fever epizootics are confirmed based on laboratory evidence of YFV infection in specimens collected from affected non-human primates or mosquitos. For rapid implementation of control measures, suspected YF epizootics are classified based on epidemiologic linkage, when four or more non-human primate deaths are identified in areas with evidence of YFV circulation, 10 or more dead animals are found in bordering areas or non-human primate die-offs occur in environments similar to those with evidence of YFV circulation. Evidence of YFV circulation included probable locations of infection of confirmed human YF cases, confirmed YF epizootics or YFV detection in mosquitoes collected during case or epizootic investigations.

Passive surveillance for epizootic disease in non-human primates is conducted as previously described [7]. Briefly, sightings of sick or dying monkeys and discovery of non-human primate carcasses were reported to the passive surveillance system through municipal or state health authorities and investigated by municipal health departments.

Active surveillance for evidence of YFV circulation included capture of forest-dwelling primates for collection of blood and sera, and collection of mosquitoes for identification and virus isolation [19]. Active surveillance was employed following investigations of non-human primate deaths when biological specimens could not be obtained or in “silent” areas with no information about presence of YFV or antibodies in non-human primate populations.

### Outbreak response

Enhanced surveillance was conducted in areas bordering those with evidence of YFV circulation or similar environmental features. Enhanced surveillance included active case finding, immediate investigation of non-human primate deaths and expedited specimen processing. Findings of non-human primate carcasses in other municipalities triggered control vaccination of the human population within a 2 km radius for events in a rural area or 300 m in urban areas pending laboratory confirmation of the presence of YFV. During the outbreak response, YF vaccination was recommended immediately from 9 months of age in municipalities with evidence of YFV circulation (“affected areas”) and all the surrounding municipalities which have borders with municipalities with confirmed YFV circulation (“expanded areas”).

### Classification of human YF cases

The suspected case definition for human YF was a patient with sudden onset of fever accompanied by jaundice or hemorrhage. Patients with recent YF vaccination were investigated for possible adverse events of vaccination, including YF vaccine-associated acute viscerotropic disease. Suspected cases with laboratory evidence of YF infection or exposure during the incubation period to affected areas without an alternative diagnosis were classified as confirmed cases. Laboratory evidence of YF infection included virus isolation [20], viral antigen identification [21], [22], detection of YF specific immunoglobulin M (IgM) by immunoassay [23] or hemagglutination inhibition tests [20]. The diagnosis of YF was discarded in suspect cases without laboratory evidence of YF infection or exposure to affected areas, or with confirmed diagnosis of another illness.

During investigations of reported deaths of non-human primates, field teams rapidly assessed vaccination coverage among residents of surrounding areas and carried out active searches for suspected cases of YF among symptomatic individuals.

The probable place of infection for human cases was defined as an area with epidemiological evidence of YFV circulation, where the human case was exposed during the incubation period.

### Data collection and analysis

For municipalities in which vaccination against YF was recommended as of July 5, 2009, we calculated the number of days from first report of deaths among non-human primates to laboratory confirmation of YFV, date of vaccine recommendation or onset of illness for confirmed human YF cases. We defined as timely vaccine recommendations within two weeks (14 days) of first reported deaths among non-human primates. We excluded one human case identified more than one year after disease occurrence during review of surveillance data.

Data on the number of YF vaccine doses administered in each municipality by month during the outbreak period were obtained from the Rio Grande do Sul state immunization program. Administrative estimates of YF vaccine coverage were obtained by dividing the cumulative number of doses administered (including doses administered prior to the outbreak from January, 2001 through October, 2008) by the total population of municipalities with YF vaccine recommendation.

## Results

Prior to the beginning of the outbreak in October, 2008, vaccination against YF was recommended in 52 municipalities, with a total population of 531,163. By June, 2009, after eight months of YFV spread, YF vaccination was recommended in 293 municipalities, with a total population of 6.9 million inhabitants (Table 1).

**Table 1:** Expansion of yellow fever vaccination recommendations, Rio Grande do Sul, Brazil, 2001—2009.

	Newly implemented YF vaccine recommendations			Area with YF vaccine recommendation			
Time period	No. municipalities	Population	Vaccine doses applied	No. municipalities (% total)	Population (% total)	Cumulative YF vaccine doses applied	% target population
Jan, 2001–Oct, 2008	-	-	-	52 (11)	531,163 (5)	613,494	>100
Nov–Dec, 2008	35	473,431	212,705	87 (18)	1,004,594 (9)	917,845	91.4
Jan, 2009	47	702,895	330,714	134 (27)	1,704,873 (16)	1,472,992	86.4
Feb, 2009	7	122,236	16,319	141 (28)	1,827,109 (17)	1,680,569	92
Mar, 2009	41	596,347	70,159	182 (37)	2,423,456 (23)	1,854,122	76.5
Apr, 2009	108	4,432,472	569,372	290 (59)	6,855,928 (64)	3,078,124	44.9
May, 2009	-	-	-	290 (59)	6,855,928 (64)	4,057,823	59.2
Jun, 2009	-	-	-	290 (59)	6,855,928 (64)	4,211,432	61.4
July, 2009	3	105,027	18,445	293 (59)	6,960,955 (65)	4,241,585	60.9

Number of municipalities with yellow fever vaccine recommendations and municipalities in which vaccination against yellow fever was recommended during a yellow fever outbreak, by time period, number of municipalities affected and resident population.

**Figure 2:**
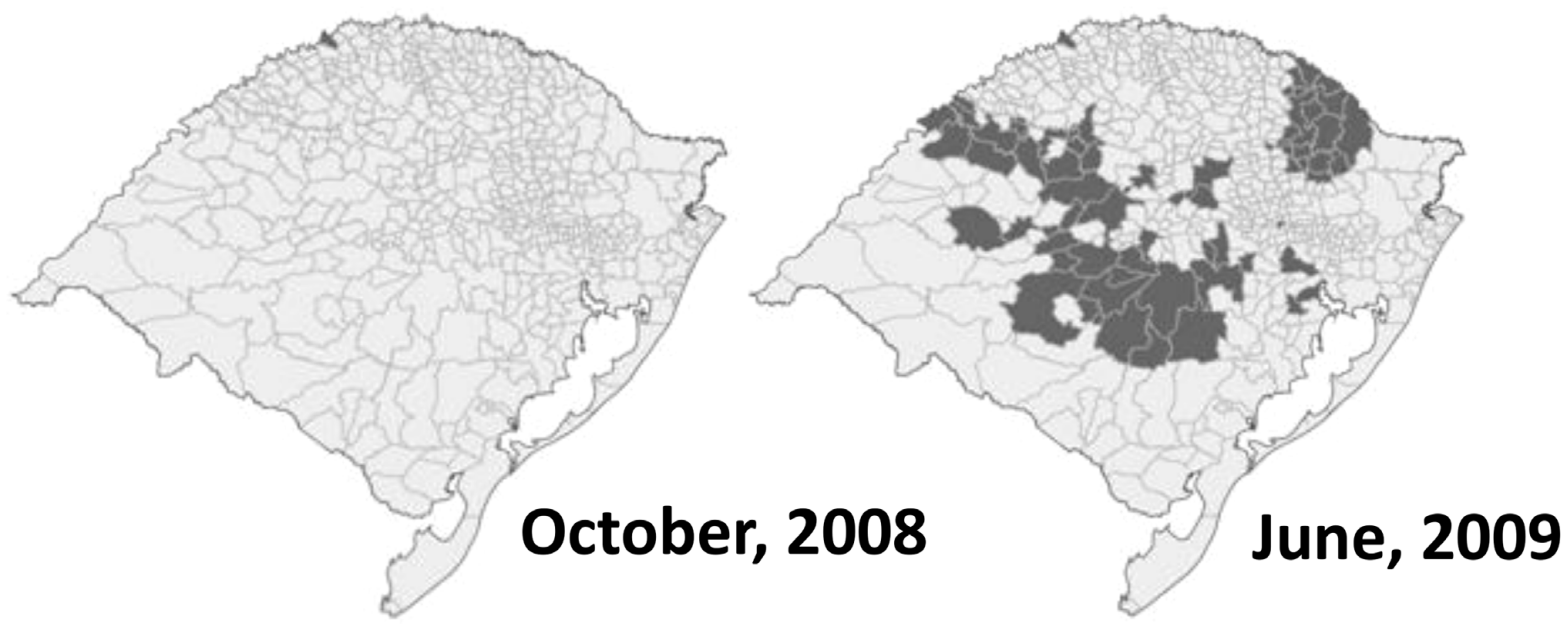
Temporal and geographic spread of yellow fever epizootics involving deaths of non-human primates, Rio Grande do Sul, Brazil, October, 2008 to June, 2009. (Shading corresponds to municipalities with confirmed yellow fever epizootics in non-human primates).

Of the 293 municipalities in which yellow fever vaccination was recommended by July, 2009, 67 (23%) were classified as “affected areas” (based on confirmed YFV circulation during the outbreak period) and 226 (77%) as “expanded areas” (Figure 2). Of these, 61 (21%) municipalities reported monkey deaths without laboratory evidence of YFV circulation and 165 (56%) were adjacent to municipalities with YFV circulation but had no reports of monkey deaths during the outbreak period. In 26 additional municipalities, epizootics were reported but laboratory and environmental investigations did not suggest YFV circulation and vaccination was not recommended.

**Figure 3:**
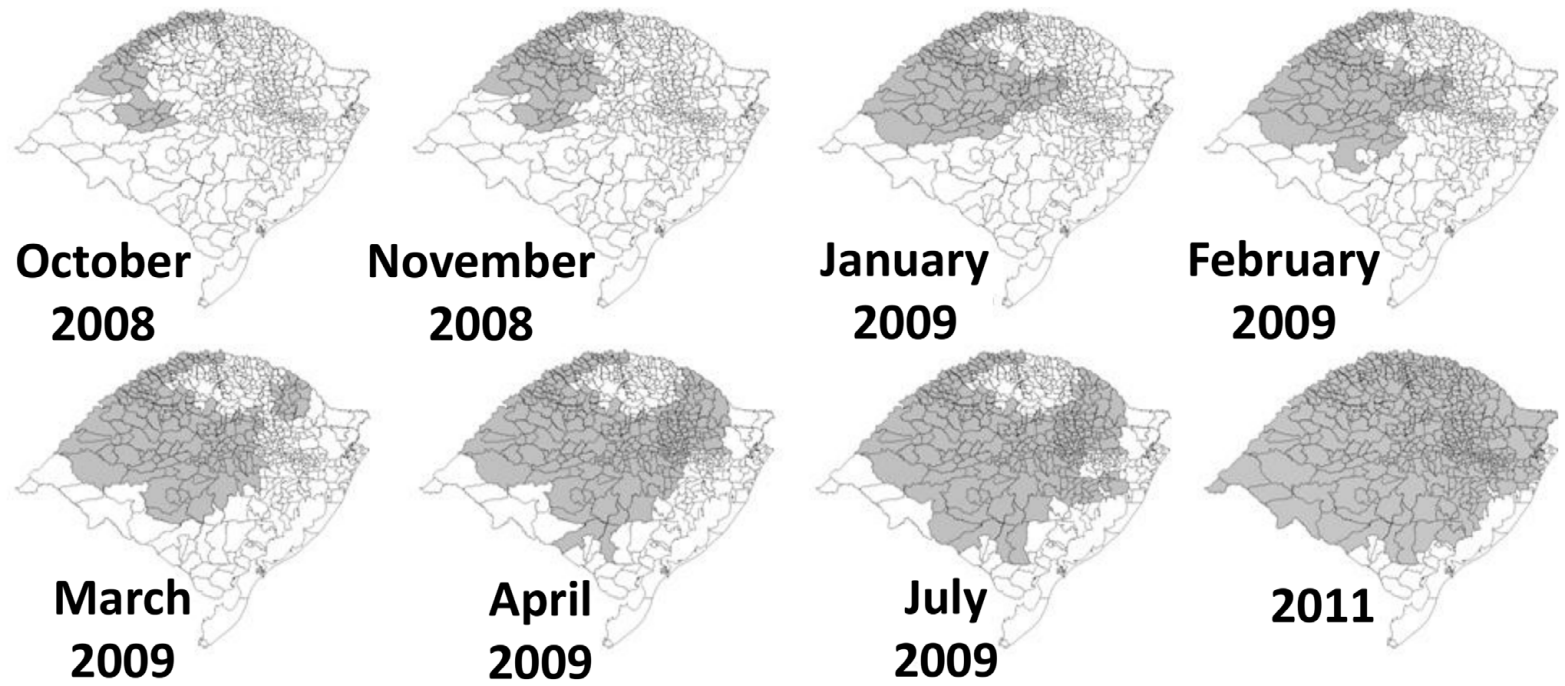
Expansion of yellow fever vaccination recommendations, Rio Grande do Sul, Brazil, 2008—2011. Municipalities in which YF vaccination was recommended (shaded area) by timing of recommendation.

Between November 30, 2008 and July 5, 2009, vaccination against YF was initiated in 241 municipalities (Figure 3). Among 67 municipalities with confirmed YFV circulation, implementation of YF vaccine recommendations was considered timely in 39 (58%): YF vaccine was recommended before the outbreak in 23 municipalities (11 with vaccination since 2001) while in 16, recommendations were implemented within 2 weeks of the first report of epizootic activity. In 28 (42%) of 67 municipalities with confirmed YFV circulation, vaccine recommendations were implemented more than 2 weeks (range 15 to 138 days) after first reported epizootic activity.

During the 9-month period, a total of 118 suspected human cases of YF were reported from 59 municipalities: 21 (18%) were confirmed based on laboratory and epidemiologic evidence, while YF was discarded in 97 (82%) without laboratory evidence of YF infection. Case-fatality among confirmed YF cases was 43% (9 of 21). None of the confirmed cases had documented history of YF vaccination. All confirmed human cases had been exposed to forested areas and, consequently, to the presence of sylvatic vectors, including *Haemagogus leucocelaenus*. Enhanced surveillance did not identify the presence of other mosquito vectors, including *H. spegazzinii*, *H. janthinomys* or *Sabethes sp.* Specimens from human cases were collected an average of 10 days (range, 2 to 17 days) after reported onset of symptoms and YFV infection was confirmed by laboratory testing from 13 to 53 days after symptom onset.

Nine municipalities were identified as probable locations of infection for 21 confirmed human YF cases. Of these, deaths of non-human primates were reported in eight (89%) municipalities 12 days to two months prior to onset of illness in persons with confirmed YF (Table 2). In two municipalities in the northwestern part of the state (each the probable location of infection for one human case) in which vaccination had been recommended since 2001, one case occurred in a recently arrived resident and one in a visitor to the area. Although in both cases family members reported being vaccinated, neither case patient was aware of YF vaccine recommendations in the area. In four other municipalities in the northwestern part of the state, YF vaccination was recommended within 7 days of the first report of deaths among non-human primates, before results of laboratory testing confirmed YFV epizootics. In two municipalities in the central part of the state (Santa Cruz do Sul and Vera Cruz), probable locations of infection for 11 human cases, vaccination began more than two months after deaths of non-human primates were first reported. Specimens collected in the municipality following initial investigations tested negative for the presence of YFV (Table 2).

**Table 2:** Timeliness of yellow fever vaccine recommendations.

Municipality	Date of first reported epizootic	Days from report to specimen collection	Days from report to laboratory confirmation	Date of vaccine recommendation	Number of confirmed human cases	Period of illness onset
Augusto Pestana	28 Nov 08	0	13	3 Dec 08	1	10 Dec 08
Santo Angelo	NR	NA	NA	3 Dec 08	3	18 Dec 08–14 Jan 09
Pirapo	27 Nov 08	5	25	2001	2	29 Dec 08–1 Jan 09
Joia	1 Dec 08	0	29	3 Dec 08	1	30 Dec 08
Bossoroca	1 Dec 08	1	22	2001	1	31 Dec 08
Ijui	28 Nov 08	0	14	3 Dec 08	1	5 Jan 09
Espumoso	31 Dec 08	6	Inc	7 Jan 09	1	12 Jan 09
Santa Cruz do Sul	21 Jan 09	44*	64	25 Mar 09	7	9 Mar–25 Apr 09
Vera Cruz	21 Jan 09	65*	82	25 Mar 09	4	15 Mar–26 Mar 09

Dates of reported deaths or epizootics among non-human primates and onset of illness for confirmed human yellow fever cases in nine municipalities identified as the Probable Place of Infection for confirmed cases, Rio Grande do Sul, Brazil, October, 2008 to June, 2009.

* Note: Denotes interval (in days) from first reported epizootic to collection of YFV-positive specimens. No specimens were obtained from initial investigations on 21 January 2009 in Santa Cruz do Sul and Vera Cruz. Specimens collected on 3 and 14 February from Santa Cruz do Sul and on 19 February 2009 from Vera Cruz tested negative for the presence of YFV.

By the end of February, 2009, more than 1.6 million doses of YF vaccine had been administered in municipalities in which vaccine was recommended, with a total population of 1.8 million. Rural areas were prioritized for vaccination. By July, 2009, more than 4.2 million doses had been administered, but the total population in municipalities with YF vaccine recommendation had jumped to 6.9 million, resulting in lower estimates of administrative coverage. Estimated coverage in more populous urban areas lagged behind coverage in rural areas. By the end of 2010, administrative coverage of YF vaccine in the area where vaccination was recommended was greater than 80%. Of 67 municipalities with confirmed YF epizootics during the 2008–09 outbreak, 41 (69%) reported administrative vaccination coverage of 90% or greater, versus 124 (55%) of 226 municipalities without confirmed YF epizootics during the outbreak.

## Discussion

Our analysis of the vaccination response to a YF outbreak in southern Brazil in 2008–2009 highlights challenges of using epizootic surveillance as an early warning system for YF in humans. Delay in detection or confirmation of YF epizootics may have resulted in late initiation of vaccination strategies in several municipalities with documented YFV transmission. Reporting of suspected epizootics and vaccination activities occurred most rapidly in areas where viral circulation had been identified during epizootic activity in 2001 and 2002, where many professionals had received training on YF surveillance. However, the rapid spread of YFV to areas previously free of the virus [7], where YF vaccination was not recommended, overwhelmed public health services, affecting the timeliness of the sampling and laboratory confirmation of YFV circulation. Even when specimens were obtained, laboratory confirmation by reference laboratories of the presence of YFV required a minimum of 13 days. Immunization strategies take time to reach high coverage, and vaccine-induced immunity develops over 7–14 days [1]. Early in an outbreak, information from epizootic surveillance may be most useful to identify populations at highest risk of YFV exposure to prioritize vaccination and other prevention strategies, including personal protection.

In two municipalities that accounted for more than 50% of human cases, detection of YFV in non-human primates was delayed. Despite collection of specimens from non-human primates prior to occurrence of human cases, vaccination was not initiated until later specimens tested positive for YFV. Delayed initiation of vaccination in these municipalities where YF vaccine had not been recommended previously may have contributed to higher numbers of people infected in this area. Prevention of human YF cases requires immediate risk communication and implementation of vaccine recommendations after the first reports of epizootic activity when YF is suspected. Based on epizootic surveillance, YF vaccination was recommended in 165 municipalities in “expanded areas” without any reports of dead monkey sightings, as well as 61 municipalities in which epizootic events were not confirmed to be yellow fever. By defining outlying areas as “at risk”, authorities did not wait to confirm YFV circulation before initiating vaccination activities.

Investigation of reported monkey deaths during the outbreak provided evidence of extensive YF epizootics in non-human primates [7]. In six months, YFV circulation in monkeys spread more than 600 km from west to east, spreading from 2 municipalities with confirmed circulation of YFV to 67 municipalities [7], [17]. Prior to the epidemic, active surveillance for the presence of YFV and other arboviruses among howler monkeys (*Alouatta* sp.) identified no immunity to YFV in this population [16]. However, active surveillance identified YFV-antibodies in howler monkey captured in November, 2009, several months after the outbreak period (unpublished data). Data from the investigations of epizootic activity during this outbreak provided new information about YFV transmission in susceptible howler monkey populations. Further study is needed to understand how YFV is spread between troops of howler monkeys in areas susceptible to YFV circulation and how environmental factors influence YFV occurrence and spread.

In 2011, following the epidemic, the Rio Grande do Sul state health department in coordination with the Brazilian Ministry of Health recommended YF vaccination in an additional 169 municipalities (Figure 3), leaving only 34 (7%) of 496 municipalities in the state without vaccine recommendation. The decision to extend YF vaccination to previously unaffected municipalities was based on the rapid spread of YFV circulation in 2008/2009, environmental characteristics, population movements and discussions with YF experts and scientific advisory committees. Better communication of YF recommendations to travelers are also needed; two of the human cases were probably infected with YF virus in areas in which vaccination had been recommended since 2001, yet neither was aware of vaccine recommendations.

The purpose of surveillance for YFV infection in non-human primates is to prevent YF cases and deaths among humans. In Rio Grande do Sul, surveillance in non-human primates was unable to predict the 2008–2009 outbreak or the rapid spread of YFV transmission, although it detected YFV circulation in non-human-primates in October, 2008 before human cases occurred. Also, active surveillance prior to the outbreak identified a susceptible population of non-human primates. During the outbreak, surveillance for epizootic activity allowed health authorities to monitor virus spread and informed vaccination strategies. In addition, surveillance for epizootics in non-human primates may have averted human cases by alerting the public to the risk of YF and intensifying vaccination efforts, although the number of human cases prevented through vaccination is unknown. Unfortunately, we had different levels of response according to the preparedness of municipalities and the familiarity of the various regions of the state with this type of emergency. The epidemic of 2008–2009 hit areas of the state in which we had no information on any previous occurrence of YF. Active surveillance for YFV infection in non-human primates in the state also developed capacity by training over 300 health professionals from Rio Grande do Sul and other Brazilian states, leading to improved reporting and investigation of epizootic events. Continued monitoring and evaluation of surveillance for YF in non-human primates in Brazil is essential to improve performance. Communication strategies to inform persons at risk of exposure to sylvatic yellow fever may also encourage reporting of epizootic events, to improve rapid detection and response to future YF outbreaks.
